# Supplementary material for: Inhalable Polymeric Nanoparticles for Pulmonary Delivery of Antimicrobial Peptide SET-M33: Antibacterial Activity and Toxicity In Vitro and In Vivo
Source: Pharmaceutics. 2022 Dec 20;15(1):3. doi: 10.3390/pharmaceutics15010003 (PMC9863998; doi:10.3390/pharmaceutics15010003)
Supplement: Supplementary file 1 [file pharmaceutics-15-00003-s001.zip › pharmaceutics-1993138-supplementary.pdf]

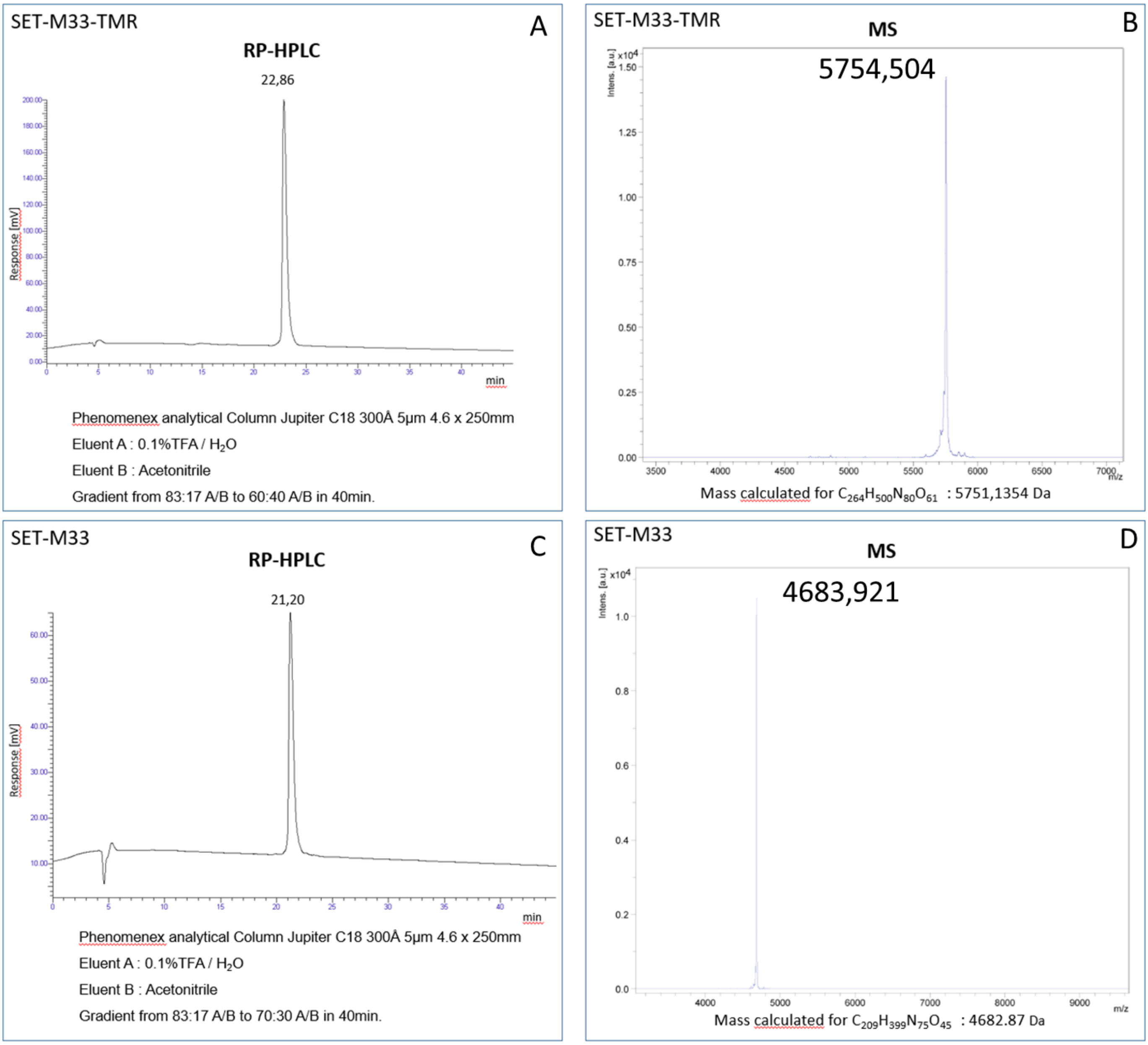

Figure S1. HPLC and Mass Spectrometry (MS) profiles of SET-M33 conjugated with tetramethyl-rhodamine (SET-M33-TMR) and free SET-M33. A and C, reverse phase HPLC profiles of SET-M33-TMR (A) and SET-M33 (C) with test method (below) and retention times (above the peaks). B and D, MS profiles of SET-M33-TMR (B) and SET-M33 (D) with the indication of revealed (above the main peak) and calculated MW (below the graph).
